# Praziquantel, Mefloquine-Praziquantel, and Mefloquine-Artesunate-Praziquantel against *Schistosoma haematobium*: A Randomized, Exploratory, Open-Label Trial

**DOI:** 10.1371/journal.pntd.0002975

**Published:** 2014-07-17

**Authors:** Jennifer Keiser, Kigbafori D. Silué, Lukas K. Adiossan, Nicaise A. N'Guessan, N'Chou Monsan, Jürg Utzinger, Eliézer K. N'Goran

**Affiliations:** 1 Department of Medical Parasitology and Infection Biology, Swiss Tropical and Public Health Institute, Basel, Switzerland; 2 University of Basel, Basel, Switzerland; 3 Unité de Formation et de Recherche Biosciences, Université Félix Houphouët-Boigny, Abidjan, Côte d'Ivoire; 4 Centre Suisse de Recherches Scientifiques en Côte d'Ivoire, Abidjan, Côte d'Ivoire; 5 Hôpital Général de Taabo, Taabo Cité, Côte d'Ivoire; 6 Institut National de la Santé Publique, Abidjan, Côte d'Ivoire; 7 Department of Epidemiology and Public Health, Swiss Tropical and Public Health Institute, Basel, Switzerland; Ministère de la Santé Publique et de la Lutte contre les Endémies, Niger

## Abstract

**Background:**

Treatment and morbidity control of schistosomiasis relies on a single drug, praziquantel. Hence, there is a pressing need to develop additional therapeutics against schistosomiasis. The antimalarial drug mefloquine shows antischistosomal activity in animal models and clinical trials, which calls for further investigations.

**Methodology:**

We comparatively assessed the efficacy and tolerability of the following treatments against *Schistosoma haematobium* in school-aged children in Côte d'Ivoire: (i) praziquantel (40 mg/kg; standard treatment); (ii) mefloquine (25 mg/kg) combined with praziquantel (40 mg/kg); and (iii) mefloquine-artesunate (3× (100 mg artesunate +250 mg mefloquine)) combined with praziquantel (40 mg/kg) (treatments administered on subsequent days). Two urine samples were collected before, and on days 21–22 and 78–79 after the first dosing.

**Principal Findings:**

Sixty-one children were present on all examination time points and had complete datasets. No difference in efficacy was observed between the three treatment groups on either follow-up. On the 21–22 day posttreatment follow-up, based on available case analysis, cure rates of 33% (95% confidence interval (CI) 11–55%), 29% (95% CI 8–50%), and 26% (95% CI 5–48%) were observed for praziquantel, mefloquine-artesunate-praziquantel, and mefloquine-praziquantel, respectively. The corresponding egg reduction rates were 94% and above. On the second follow-up, observed cure rates ranged from 19% (praziquantel) to 33% (mefloquine-artesunate-praziquantel), and egg reduction rates were above 90%. Praziquantel monotherapy was the best tolerated treatment. In the mefloquine-artesunate-praziquantel group, adverse events were reported by 91% of the participants, and in the mefloquine-praziquantel group, 95% experienced adverse events. With the exception of abdominal pain at moderate severity, adverse events were mild.

**Conclusions/Significance:**

The addition of mefloquine or mefloquine-artesunate does not increase the efficacy of praziquantel against chronic *S. haematobium* infection. Additional studies are necessary to elucidate the effect of the combinations against acute schistosomiasis.

## Author Summary

The antimalarial drug mefloquine shows activity against blood flukes that cause the disease schistosomiasis. In animal studies it has been found that a mefloquine-praziquantel combination kills blood flukes more effectively than praziquantel alone. Combining praziquantel with another drug might therefore increase efficacy, broaden the spectrum of activity, and delay the development of drug resistance. We designed a study in Ivorian school children to assess the efficacy and tolerability of mefloquine and mefloquine-artesunate combined with praziquantel against the blood fluke *Schistosoma haematobium*. The administration of the antimalarials and praziquantel was spaced by a day. Treatment outcomes were assessed twice, on days 21–22 and 78–79 after the first dosing to determine the effect against adult and juvenile *S. haematobium*, respectively. At both follow-ups, high reduction in the intensity of infection (egg reduction rates of 94–96%), but low cure rates (26–33%) were observed in the three treatment groups. Adverse events were common, particularly in children treated with mefloquine-praziquantel and mefloquine-artesunate-praziquantel. Our study suggests that the addition of mefloquine and mefloquine-artesunate to praziquantel has no benefit in the treatment of chronic *S. haematobium* infection. However, further investigations are warranted to evaluate the effect of combination therapy on juvenile flukes and longer-term morbidity profiles.

## Introduction

Schistosomiasis is a neglected tropical disease caused by a chronic infection with blood-dwelling parasitic flatworms of the genus *Schistosoma* [1]. More than 230 million people in the tropics and subtropics are infected and the global burden of schistosomiasis is estimated at 3.3 million disability-adjusted life years (DALYs) [2], [3]. The main strategy to control schistosomiasis is preventive chemotherapy that is the periodic administration of the antischistosomal drug praziquantel to populations at-risk of morbidity, most importantly school-aged children [4]–[6]. Although no clinically relevant resistance to praziquantel has been documented thus far, reliance on a single drug is a risky endeavor [7], [8]. Moreover, praziquantel is quite ineffective against the young developing stages of schistosomes [9], [10]. To address this inherent shortcoming of praziquantel, treatment of acute infections will have to be repeated or postponed until worms will have matured [1]. It is thus essential to find additional therapeutics against schistosomiasis, ideally compounds that are active against all stages of the parasite.

There is presently no other broad-spectrum antischistosomal drug available and the drug development pipeline is empty. Against this background and taking into consideration scarce resources for research and development of neglected tropical diseases, repurposing of drugs that are already approved for human use is a promising strategy [11]. Indeed, such a strategy is more rapid, less risky, and less costly than developing new drugs [12], [13].

Since 2008, the antimalarial drug mefloquine is undergoing detailed *in vitro*, *in vivo*, and clinical investigation for its trematocidal properties. For example, in the *Schistosoma mansoni*-mouse model, mefloquine exhibited high worm burden reductions following single-dose regimen against juvenile and adult schistosomes [14]–[16]. Mefloquine also revealed a high activity against the other two major human schistosome species, *S. haematobium* [17] and *S. japonicum* [14], [18]. It was therefore concluded that mefloquine has a similarly broad spectrum of activity than praziquantel. In an exploratory trial in Côte d'Ivoire, a mefloquine-artesunate combination showed a moderate cure rate (61%) and high egg reduction rate (96%) in school-aged children infected with *S. haematobium* [19]. Recently, mefloquine, used as intermittent preventive therapy against malaria in pregnancy (IPT_p_), showed high egg reduction rates in women with a concomitant *S. haematobium* infection [20]. However, combination therapy with mefloquine and praziquantel, which showed high worm burden reductions in laboratory animals [21], has not yet been studied in *Schistosoma*-infected patients.

The aim of the current study was to assess the efficacy and tolerability of mefloquine and mefloquine-artesunate combined with praziquantel against *S. haematobium* in school-aged children. Since prior *in vivo* studies revealed synergistic effects when mefloquine and praziquantel were administered on subsequent days, and drug interaction between mefloquine and praziquantel have not been studied before, drug administration of the antimalarials and praziquantel was spaced by a day. For comparison, one group of children was treated with praziquantel only, using the current standard dose of 40 mg/kg. Treatment outcomes were assessed twice, on days 21–22 and 78–79 after the first dosing to determine the effect against pre-patent and patent *S. haematobium* infection.

## Methods

### Ethics Statement

Ethical clearance was obtained by the ethics committee in Basel (EKBB; reference no. 70/08) and the Ministère de la Santé et de l'Hygiène Publique en Côte d'Ivoire. Parents/guardians of participating children signed a written informed consent for their children, and children assented orally. Participation was voluntary and the children were informed that they could withdraw anytime without further obligation. The trial is registered with Current Controlled Trials (ISRCTN00393859).

### Study Site

The study was carried out in Sahoua, a village in the Taabo district, located about 170 km north-west of Abidjan, the economic capital of Côte d'Ivoire. Sahoua is situated in the V-Baoulé, the transition zone between the rainforest in the South and the Savannah in the North, at the north-western edge of the Taabo health and demographic surveillance system (HDSS) [22]. The climate is tropical with the main rains occurring between April and July and in September/October. People are primarily engaged in subsistence farming (e.g., cassava, plantains, and yams), whilst cacao is the predominant cash crop. The village of Sahoua is close to the Bandama River. The inhabitants coming from neighboring countries Mali and Burkina Faso are fishers. Women perform household chores (e.g., washing dishes or clothes) at the water's edge. School-aged children are in frequent contact with the water during recreational activities (e.g., bathing and swimming).

### Study Flow

The field and laboratory work was carried out between November 2011 and February 2012. The study aim was explained and approved by the local health authorities, including the director of Taabo-Cité hospital, the district health officer of Tiassalé, the village chief, and the school director. Parents/guardians of the children provided written informed consent, while children assented orally. All school children from grade 3 (CE1) to 6 (CM2) were invited to participate in the prescreening. A total of 130 school children provided a urine sample. Urine samples were collected between 10:00 and 14:00 hours and labeled with unique identifiers. Samples were transferred to the laboratory of the Taabo-Cité hospital for macroscopic and microscopic examination of *S. haematobium*. 77 children were identified as positive and invited to participate in the study. These children were asked to provide an additional urine sample and a stool sample the next day. Those children who had complete parasitological datasets were invited for a clinical examination, which included a physical examination, weight measurement (using an electronic balance recording to the nearest 0.1 kg), assessing temperature (using battery-powered ear thermometers to the nearest 0.01°C), and a finger-prick blood sample. From the blood sample, hemoglobin concentration was determined using a portable Hemocue 301 (HemoCue AB; Ängelholm, Sweden). Additionally, thick and thin blood films were prepared on microscope slides, labeled with unique identification numbers, and air-dried.

Children were excluded if any of the following criteria were met: (i) fever (temperature ≥37.5°C); (ii) pregnancy first trimester assessed verbally; (iii) presence of any abnormal medical condition, judged by the study physician; (iv) history of acute or severe chronic disease; (v) psychiatric disorders such as epilepsy; (vi) recent use of anthelmintic or antimalarial drugs (within the past month); and (vii) weight below 20 kg. *S. haematobium*-infected children who were excluded from the study were offered praziquantel (40 mg/kg) free of charge.

Mefloquine (250 mg lactabs), and mefloquine-artesunate blisters containing 3×100 mg artesunate and 3×250 mg mefloquine were purchased from Viktoria Apotheke (Zurich, Switzerland). Praziquantel (600 mg tablets) was purchased from Inresa (Bartenheim, France). Children included in the study received one of three treatments under direct medical observation, following a computer-generated randomization code: (i) mefloquine 25 mg/kg single dose (body weight <30 kg) or a split dose spaced by 6 hours (body weight ≥30 kg) plus a single dose of praziquantel (40 mg/kg) on the next day; (ii) mefloquine-artesunate (1×100 mg artesunate and 1×250 mg mefloquine) once daily for 3 consecutive days plus a single dose of praziquantel (40 mg/kg) on treatment day 4; and (iii) praziquantel, standard single dose (40 mg/kg). Mefloquine and praziquantel were administered to the nearest half tablet according to the calculated dose per kg of body weight. All participating children received a snack shortly after drug administration. Children were kept for observation and interviewed for the presence of acute adverse events 3 hours posttreatment. In addition, adverse events were assessed 24 hours after each treatment dose (prior to the next dose for the mefloquine-artesunate-praziquantel and mefloquine-praziquantel treatment groups). Adverse events were graded (i.e., mild, moderate, severe, and life-threatening), and symptomatic relief provided if necessary.

### Laboratory Procedures

Urine samples were examined visually for macroscopic blood and then analyzed for microhematuria using reagent strips (Hemastix, Siemens Healthcare; Zurich, Switzerland). For detection of *S. haematobium* eggs, urine samples were subjected to a filtration method [19]. In brief, samples were carefully homogenized and 10 ml of urine pressed through a 13-mm diameter filter with 25 µm pores (Sefar AG; Heiden, Switzerland). The filters were placed on microscope slides, a drop of Lugol's solution added before examination under a microscope at a magnification of ×100 by two experienced technicians. Slides were re-examined by a senior technician in case of differing results among the two technicians.

Stool specimens were subjected to duplicate Kato-Katz thick smears, using standard 41.7 mg templates [23] and examined under a microscope. The number of *S. mansoni*, *Ascaris lumbricoides*, *Trichuris trichiura*, hookworm, and other helminth eggs were counted and recorded for each species separately. Additionally, approximately 2 g of stool was preserved in sodium acetate-acetic acid-formalin (SAF), and processed with an ether-concentration method [24]. Samples were examined microscopically at a magnification of ×100 for helminths, and at a magnification of ×400 for intestinal protozoa (e.g., *Blastocystis hominis*, *Chilomastix mesnili*, *Endolimax nana*, *Entamoeba coli*, *Entamoeba hartmanni*, *Entamoeba histolytica/E. dispar*, *Giardia intestinalis*, and *Jodamoeba bütschlii*).

Thick and thin blood films were stained with Giemsa, and prepared and read as described elsewhere [19]. Parasite counts were documented as the number of *Plasmodium* per µl of blood, assuming a standard count of 8,000 white blood cells per µl of blood.

### Sample Size, Statistics, and Outcome Measures

By definition, pilot studies are conducted to serve as a starting point for further studies and are primarily intended to yield information about the feasibility and implementation possibilities of novel treatments. Thus, the choice of an adequate sample size for a pilot study is mainly based on practical considerations of the pilot trial rather than on statistical sample size calculations [25]. Allowing for up to 50% drop-outs, we aimed for 23–25 children per treatment arm. Data were double entered into Excel and Access (Microsoft 2010), cross-checked, and analyzed using Stata version 10.1 (StataCorp.; College Station, United States of America) and Statsdirect version 2.7.9 (Statsdirect, Chesire, United Kingdom).

All children with primary endpoint data were included in the analysis (available case analysis). *S. haematobium* egg counts from the two urine samples were averaged for each child (arithmetic mean (AM)) and the AM and geometric mean (GM) egg count for each treatment group calculated. Cure rate (percentage of children excreting no *S. haematobium* eggs at the posttreatment follow-ups (i.e., 21–22 and 78–79 days after drug administration) among parasitological-confirmed children at baseline) and egg reduction rate (reduction of AM and GM egg count among *S. haematobium*-positive children posttreatment compared to the respective AM or GM pretreatment) were calculated. Bootstrap resampling method with 10,000 replicates was used to calculate 95% confidence intervals (CIs) for egg reduction rates of GM [26]. Differences in egg reduction rates were determined under the assumption that non-overlapping CIs indicate statistical significance. To test whether there was an association between cure rates and dose, the actual doses administered were determined and analyzed using logistic regression. To compare baseline and follow-up parameters, Mann-Whitney U test and Wilcoxon matched pairs test were used, as appropriate. Pearson's χ^2^ was used to compare the proportion of reported adverse events between treatment arms.

## Results

### Adherence, Participants, and Baseline Parameters

**Figure 1:**
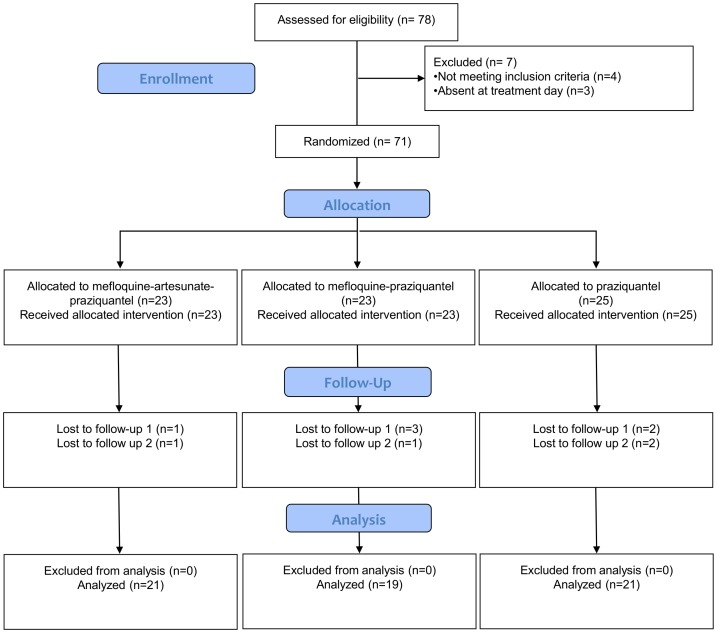
Consort flowchart. Study enrollment, allocation, follow-up, and analysis.

Overall, 71 *S. haematobium*-infected children were randomized to the three treatment arms (Figure 1). Ten children were lost at the first or second follow-up, mainly because of travels at the time of the surveys. Demographic and clinical baseline characteristics of the 61 children included in the available case analysis are summarized in Table 1. Treatment groups were well balanced in terms of age (mean age: 10.4–10.9 years), weight (mean weight: 28.0–29.1 kg), and height (mean height: 138.8–139.9 cm). However, more boys (n = 47) than girls (n = 14) participated in the trial. Hemoglobin values were in the normal range.

**Table 1:** Demographic and laboratory baseline characteristics of *S. haematobium*-infected children treated with mefloquine-artesunate plus praziquantel, mefloquine plus praziquantel, and praziquantel.

Parameter	Treatment group		
	Artesunate-mefloquine plus praziquantel (n = 21)	Mefloquine plus praziquantel (n = 19)	Praziquantel (n = 21)
Males/females (no.)	15/6	16/3	16/5
Mean (±SD) age, years	10.4 (2.8)	10.7 (2.7)	10.9 (2.4)
Mean (±SD) weight, kg	29.1 (8.1)	29.1 (6.0)	28.0 (5.5)
Mean (±SD) height, cm	139.5 (11.8)	139.5 (10.6)	138.8 (9.9)
Mean (±SD) hemoglobin (g/dl)	11.6 (1.0)	12.0 (0.7)	12.0 (1.0)
*S. haematobium* infection			
GM eggs/10 ml urine	61.8	45.2	45.9
AM eggs/10 ml urine	177.7	140.7	89.1
Range	1–1,198	1–1,219	1–792
Number (%) of light infection**	8 (38)	8 (42)	9 (43)
Number (%) of heavy infection	13 (62)	11 (58)	12 (57)
Urinary analysis, number (%) of children with			
Microhematuria (by reagent strip)			
Negative	2 (10)	4 (21)	3 (14)
Trace positive	3 (14)	2 (11)	6 (29)
Positive	16 (76)	13 (68)	12 (57)
Macrohematuria (by visual inspection)			
Negative	1 (5)	4 (21)	6 (29)
Trace positive	4 (19)	5 (26)	8 (38)
Positive	16 (76)	10 (53)	7 (33)
Malariometric indices			
Number (%) of children with *Plasmodium*	21 (100)	18 (100)*	17 (81)
Parasitemia (mean density/µl of blood)	4816	1967	3249
Number (%) of children infected with soil-transmitted helminths	2 (10)	1 (5)	0 (0)
Number (%) of children infected with intestinal protozoa			
*Endolimax nana*	6 (29)	10 (53)	6 (29)
*Entamoeba coli*	12 (57)	7 (37)	8 (38)
*Giardia intestinalis*	4 (19)	6 (32)	3 (14)
*Chilomastrix mesnili*	2 (10)	2 (11)	0
*Blastocystis hominis*	3 (14)	3 (16)	0
*Entamoeba histolytica*/*E. dispar*	4 (19)	2 (11)	2 (10)
*Jodamoeba bütschlii*	0	4 (21)	0

* 18 malaria slides.

** <50 eggs/10 ml of urine.

Most of the included children suffered from heavy *S. haematobium* infection, as defined by ≥50 *S. haematobium* eggs per 10 ml of urine (n = 36, 59%). No difference was observed in infection intensity between the three treatment groups; the GM of *S. haematobium* eggs per 10 ml of urine ranged from 45.2 to 61.8. Macroscopic examination of urine revealed visible blood in 33 samples (46%). Indirect screening approaches based on reagent strips to detect microhematuria and proteinuria, revealed prevalences of 82% and 85%, respectively. Most children were coinfected with *Plasmodium falciparum*. No infection with *S. mansoni* was diagnosed. Coinfections with hookworm were observed in three children. Intestinal protozoa infections were common; *Endolimax nana* and *Entamoeba coli* were the predominant species in all treatment groups.

### Efficacy against *S. haematobium* and Concomitant Parasitic Infections

Our results showed no difference in cure rates against *S. haematobium* among the three treatment arms, at neither treatment follow-up. At the first follow-up 21–22 days posttreatment, we observed low cure rates; namely, 26% (95% CI 5–48%) for mefloquine plus praziquantel, 29% (95% CI 8–50%) for mefloquine-artesunate plus praziquantel, and 33% (95% CI 11–55%) for praziquantel monotherapy (Table 2). Cure rates were higher among children with light-intensity *S. haematobium* infections (38–67%). Cure rates determined with indirect screening approaches by visual inspection of urine for macrohematuria and reagent strip testing for microhematuria were as high as 71%.

**Table 2:** Effect of artesunate-mefloquine plus praziquantel, mefloquine plus praziquantel, and praziquantel on *S. haematobium* and concomitant co-infections at the first treatment follow-up.

	Treatment		
Parameter	Artesunate-mefloquine plus praziquantel (n = 21)	Mefloquine plus praziquantel (n = 19)*	Praziquantel (n = 21)
Mean (±SD) hemoglobin (g/dl)	12.7 (1.2)	12.6 (1.0)	12.4 (1.2)
*Schistosoma haematobium* infection			
Cure rate (%) (95% CI)	29 (8 to 50)	26 (5 to 48)	33 (11 to 55)
Number (%) of children cured with light infections	3 (38)	4 (50)	6 (67)
Number (%) of children cured with heavy infections	3 (23)	1 (9)	1 (8)
Geometric mean (eggs/10 ml of urine)	2.3	2.4	2.7
Egg reduction rate, % (95% CI)	96 (93 to 99)	95 (91 to 98)	94 (86 to 97)
Arithmetic mean (eggs/10 ml of urine (range))	6.4 (0–126)	5.5 (0–54)	9.0 (0–144)
Egg reduction rate, %	96	96	90
Urinary analysis, number (%) of children with			
Microhematuria (by reagent strip)			
Negative	15 (71)	13 (68)	15 (71)
Trace positive	5 (24)	6 (32)	5 (24)
Positive	1 (5)	0 (0)	1 (5)
Macrohematuria (by visual inspection)			
Negative	13 (61.9)	10 (52.6)	15 (71.4)
Trace positive	4 (19)	4 (21)	3 (14)
Positive	4 (19)	5 (26)	3 (14)
Malariometric indices			
Number (%) of children with *Plasmodium*	0 (0)	1 (5)	18 (86)
Parasitemia (mean density/µl of blood)	0 (0)	80	711
Co-infections (number (%) of infected children)			
Hookworm	3 (14)	2 (11)	11 (52)
Intestinal protozoa			
*Endolimax nana*	5 (24)	3 (17)	10 (48)
*Entamoeba coli*	12 (57)	10 (56)	8 (38)
*Giardia intestinalis*	5 (24)	2 (11)	0 (0)
*Entamoeba histolytica/E. dispar*	6 (29)	5 (28)	1 (5)
*Blastocystis hominis*	1 (5)	1 (6)	1 (5)
*Chilomastrix mesnili*	5 (24)	0 (0)	2 (10)
*Jodamoeba bütschlii*	4 (19)	3 (17)	2 (10)

*18 SAF samples available.

No significant association between cure rates and exact dose was observed. In the praziquantel treatment arm, the exact dose of praziquantel administered ranged from 33 to 45 mg/kg; in the mefloquine-praziquantel treatment arm, the exact dose of mefloquine ranged from 22 to 25 mg/kg and that of praziquantel from 33 to 45 mg/kg; in the mefloquine-artesunate-praziquantel treatment arm, the exact dose of mefloquine ranged from 13 to 36 mg/kg, that of artesunate from 5.4 to 14.2 mg/kg, and the administered dose of praziquantel was 33–43 mg/kg.

High egg reduction rates (94–96% based on the GM eggs per 1 g of stool (EPG)) were observed for the three treatments against *S. haematobium* at the first follow-up. Additionally, all children treated with mefloquine-artesunate plus praziquantel were cured from *Plasmodium* infections. Seventeen out of 18 children treated with mefloquine plus praziquantel had a negative laboratory diagnosis of *Plasmodium*. The two groups treated with antimalarials had significantly higher hemoglobin values (p<0.05; mean increase of 0.57 g/dl hemoglobin in mefloquine-praziquantel and 1.15 g/dl in mefloquine-artesunate plus praziquantel treated children) in contrast to children treated with praziquantel alone (mean increase of hemoglobin 0.36 g/dl). As expected, no effect on concomitant *Plasmodium* infection was observed in children who were treated with praziquantel singly. The prevalence of intestinal protozoa infection was similar at baseline and the first treatment follow-up.

At the second follow-up 78–79 days posttreatment, cure rates ranged from 19% (95% CI 1–37%) (praziquantel) to 33% (95% CI 11–55%) (mefloquine-artesunate plus praziquantel) (Table 3). No difference was observed between the three treatment arms and cure rates. Additionally, no difference was observed between cure rates at the first and second follow-up. Egg reduction rates were high (92–94% based on the GM). Visual inspection and reagent strip analysis resulted in cure rates of 53–67% and 47–62%, respectively. Eleven children in the antimalarial treatment groups had re-acquired a malaria infection. Hemoglobin levels in children treated with mefloquine-artesunate plus praziquantel remained significantly higher compared to baseline values (12.9 *versus* 11.6 g/dl; p<0.001). In addition, infection with *E. coli* were most commonly observed (37 children), followed by *E. nana* and hookworm infections (24 and 17 children, respectively).

**Table 3:** Effect of artesunate-mefloquine plus praziquantel, mefloquine plus praziquantel, and praziquantel on *S. haematobium* and concomitant co-infections at the second treatment follow-up.

Parameter	Treatment		
	Artesunate-mefloquine plus praziquantel (n = 21)	Mefloquine plus praziquantel (n = 19)	Praziquantel (n = 21)
Mean (±SD) hemoglobin (g/dl)	12.9 (0.9)	12.3 (1.1)	12.0 (1.3)
*Schistosoma haematobium*			
Cure rate (%) (95% CI)	33 (11 to 55)	21 (1 to 41)	19 (1 to 37)
Number (%) of children cured with light infections	4 (50)	2 (25)	3 (33)
Number (%) of children cured with heavy infections	2 (15)	2 (18)	1 (8)
Geometric mean (eggs/10 ml of urine)	3.7	2.7	3.5
Egg reduction rate, % (95% CI)	94 (81 to 99)	94 (86 to 98)	92 (83 to 96)
Arithmetic mean (eggs/10 ml of urine (range))	11.2 (0–56)	4.7 (0–22)	10.9 (0–178)
Egg reduction rate, %	94	97	88
Urinary analysis, no (%) of children with			
Microhematuria (by reagent strip)			
Negative	13 (62)	9 (47)	11 (52)
Trace positive	4 (19)	9 (47)	9 (43)
Positive	4 (19)	1 (5)	1 (5)
Macrohematuria (by visual inspection)			
Negative	12 (57)	10 (53)	14 (67)
Trace positive	7 (33)	9 (47)	6 (19)
Positive	2 (10)	0 (0)	1 (5)
Malariometric indices			
Number (%) of children with *Plasmodium*	4 (19)	7 (37)	13 (62)
Parasitemia (mean density/µl of blood)	1,620	1,949	2,028
Co-infections (number (%) of infected children)			
Hookworm	4 (19)	3 (16)	10 (48)
Intestinal protozoa			
*Endolimax nana*	9 (43)	8 (42)	7 (33)
*Entamoeba coli*	12 (57)	15 (79)	10 (48)
*Giardia intestinalis*	4 (19)	5 (26)	3 (14)
*Entamoeba histolytica/E. dispar*	1 (5)	4 (21)	2 (10)
*Blastocystis hominis*	0 (0)	1 (5)	3 (14)
*Chilomastrix mesnili*	0 (0)	2 (11)	1 (5)
*Jodamoeba bütschlii*	1 (5)	1 (5)	4 (19)

### Adverse Events

During the clinical examination at baseline 20 of the 61 participating children reported symptoms, mainly headache and abdominal pain. The number of children experiencing adverse events, stratified by treatment arm at each of the two treatment follow-ups, is summarized in Table 4. Table 5 presents the number of specific mild and moderate adverse events, observed in each treatment arm assessed at different examination time points. We did not observe any life-threatening adverse events following treatment and, with the exception of abdominal pain at moderate severity (12 children), adverse events were mild. Praziquantel and mefloquine-artesunate were significantly better tolerated than mefloquine (p<0.05), as assessed 24 hours posttreatment. Nearly all children treated with mefloquine-artesunate plus praziquantel (91%) and mefloquine plus praziquantel (95%) experienced adverse events over the four respectively two treatment days. More than half of the children (56%) stated adverse events following a single dose of praziquantel. Abdominal pain (mild and moderate episodes) was the most commonly observed adverse event in all treatment groups. Children treated with mefloquine-artesunate plus praziquantel and mefloquine plus praziquantel reported significantly more mild abdominal pain than children treated with praziquantel (p<0.05). Vomiting was also commonly reported by children treated with mefloquine-artesunate plus praziquantel (p<0.05) and mefloquine plus praziquantel. Other common adverse events in all treatment groups included vertigo, headache, and diarrhea.

**Table 4:** Number of children with adverse events among the three treatment arms, as assessed at different time points posttreatment.

Number (%) of patients with adverse events			
Time point	Artesunate-mefloquine plus praziquantel	Mefloquine plus praziquantel	Praziquantel^a^
Related symptoms before treatment	9 (43)	3 (16)	8 (38)
3 hours after first treatment	3 (14)	11 (58)	7 (44)
24 hours after first treatment	3 (14)	12 (63)*	2 (13)
3 hours after second treatment	12 (57)	9 (47)	NA
24 hours after second treatment	6 (29)	1 (5)	NA
3 hours after third treatment	11 (52)	NA	NA
24 hours after third treatment	8 (38)	NA	NA
3 hours after fourth treatment	13 (62)	NA	NA
24 hours after fourth treatment	2 (10)	NA	NA
Overall number of patients experiencing adverse events at any time point	19 (91)	18 (95)	9 (56)

NA: not applicable.

a : 16 patients participated at adverse events examinations.

* significantly different from praziquantel (p<0.05) using Pearson's χ^2^ test.

**Table 5:** Number of specific mild and moderate adverse events, stratified by treatment, as assessed at different time points posttreatment.

		No. of adverse events																
		Artesunate-mefloquine plus praziquantel									Mefloquine plus praziquantel					Praziquantel^a^		
		Hours posttreatment									Hours posttreatment					Hours posttreatment		
Type of adverse event	Grading	3	24	3	24	3	24	3	24	No. (%) of patients at any time point	3	24	3	24	No. of patients (%) at any time point	3	24	No. (%) of patients at any time point
		(first dose)		(second dose)		(third dose)		(fourth dose)			(first dose)		(second dose)					
Headache	Mild	3	2	2	0	1	0	4	0	6 (29)	6	1	0	0	6 (32)	4	1	5 (31)
	Moderate	0	0	0	0	0	0	0	0	0	0	0	0	0	0	0	0	0
Vomiting	Mild	0	0	2	2	5	3	7	0	10 (48)*	3	5	0	0	7 (37)	2	0	2 (13)
	Moderate	0	0	0	0	0	0	0	0	0	0	0	0	0	0	0	0	0
Abdominal Pain	Mild	1	1	9	4	5	5	6	2	16 (76)*	7	8	5	0	14 (74)*	4	1	4 (25)
	Moderate	0	0	1	1	3	1	2	0	5 (24)	3	1	1	0	5 (26)	2	0	2 (13)
Vertigo	Mild	0	0	2	2	3	3	2	0	8 (38)	4	3	1	0	7 (37)	4	9	4 (25)
	Moderate	0	0	0	0	0	0	0	0	0	0	0	0	0	0	0	0	0
Diarrhea	Mild	0	0	3	1	1	2	1	0	7 (33)	1	2	1	1	4 (21)	2	0	2 (13)
	Moderate	0	0	0	0	1	0	0	0	1 (5)	0	0	0	0	0	0	0	0
Nausea	Mild	0	0	1	1	3	1	0	0	4 (19)	0	0	0	0	0	0	0	0
	Moderate	0	0	0	0	0	0	0	0	0	0	0	0	0	0	0	0	0

a : 16 patients participated at adverse events examinations.

* significantly different from praziquantel (p<0.05) using Pearson's χ^2^ test.

## Discussion

Reliance on a single drug for individual treatment and community-based morbidity control of schistosomiasis – one of the most important parasitic diseases in sub-Saharan Africa – bears the risk of parasites developing resistance. No alternative antischistosomal drugs are in the development pipeline. Oxamniquine and metrifonate – two drugs that have been widely used against *S. mansoni* and *S. haematobium*, respectively – are (with the exception of oxamniquine in Brazil) no longer commercially available [27]–. A promising approach for identifying new drugs against schistosomiasis is to repurpose existing drugs that are already on the market for the treatment of other diseases. This strategy is popular in many medical fields, including tuberculosis [30], cancer [31], and malaria [32]. In fact, a recent analysis of the research and development landscape of drugs and vaccines for neglected diseases from 2000 to 2011 showed that most new drugs in this therapeutic area are repurposed versions of existing products [11].

In the present exploratory trial, we assessed whether antimalarials (mefloquine and mefloquine-artesunate) plus praziquantel have a higher efficacy than standard single-dose praziquantel. Mefloquine and mefloquine-artesunate combination were selected as combination partner for praziquantel since laboratory studies have shown synergistic effect for mefloquine-praziquantel combinations *in vitro* and *in vivo* [21]. Furthermore, stage-specific susceptibility studies have shown that, in contrast to the biphasic activity of praziquantel, juvenile worms are particularly vulnerable to mefloquine and the artemisinins [14], [33]. Hence, we hypothesized that a mefloquine-praziquantel combination has an increased spectrum of activity compared to praziquantel alone. Note that drugs were administered on consecutive days as drug interactions have not been studied to date and the treatment schedule administering the antimalarials prior to praziquantel had achieved the highest activity *in vivo* [21].

At the first posttreatment follow-up 21–22 days after drug administration, a marked reduction in the intensity of infection with high egg reduction rates (94–96%) but low cure rates (26–33%) were observed in the three treatment groups. We were surprised about the low cure rates achieved by praziquantel, although previous studies also reported low cure rates when administering praziquantel against *S. haematobium* (e.g., 40% in Cameroon [34] and 37% in Mali [35]). As described before [6], [34], these low cure rates most likely reflect that children treated with praziquantel had high infection intensities prior to drug administration. However, most prior studies have reported higher cure rates. For example, Stothard and colleagues recently reviewed the literature and meta-analyzed the data, which revealed an overall cure rate of 70% in response to a single dose of praziquantel against *S. haematobium* [6]. Unexpectedly, the co-administration of either mefloquine or mefloquine-artesunate with praziquantel showed similarly low cure rates than the paziquantel single treatment group. Our findings therefore contrast with previous studies. In Nigeria, a combination of praziquantel and artesunate (using a similar treatment schedule than in the current investigation) achieved higher cure rates and egg reduction rates compared to single praziquantel or single artesunate [36]. In addition, a previous study conducted in a nearby village, revealed a cure rate of 61% in *S. haematobium*-infected children treated with a mefloquine-artesunate combination [19]. Hence, since mefloquine and artesunate exhibit antischistosomal properties [19], we expected to observe higher cure rates combining these antimalarials with praziquantel compared to praziquantel singly. A limitation of our study is that the viability of excreted eggs [37] was not determined, and hence counts of dead eggs might have been included in the analysis, and hence our reported cure rates might underestimate the true situation.

At the second follow-up examination 78–79 days posttreatment, cure and egg reduction rates were comparably low as in the first follow-up. A slight (not significant) decrease in the estimated cure rate of praziquantel was noted (from 33% to 19%). Given the small sample size and low cure rates observed already at the first follow-up, a conclusion whether the addition of mefloquine and/or artesunate would expand the activity profile of praziquantel targeting juvenile schistosomes cannot be drawn.

As expected, praziquantel was the best tolerated treatment, perhaps explained by only one type of drug administered. Mefloquine-praziquantel and mefloquine-artesunate-praziquantel on the other hand were administered over 2 and 4 days, respectively. The adverse event rate calculated as the number of adverse events per group, divided by the person-time at risk in each group was similar among the treatment groups (data not shown). Whether adverse events following praziquantel administration in the mefloquine-artesunate-praziquantel and mefloquine-praziquantel treated children are due to praziquantel or due to the long systemic exposure of the antimalarials is not known. Similar to our previous study [19], most children treated with mefloquine and mefloquine-artesunate reported mild or moderate adverse events, mainly gastrointestinal complaints, including abdominal pain, nausea, and vomiting.

In conclusion, our results suggest that a drug combination containing mefloquine-artesunate or mefloquine has no benefit over standard praziquantel against chronic *S. haematobium* infection regarding efficacy (cure and egg reduction rate) and safety (frequency and severity of adverse events). Further studies are required to elucidate the effect of these combinations on acute schistosomiasis. There is a pressing need to develop additional antischistosomal drugs, as long as praziquantel remains efficacious against different *Schistosoma* species parasitizing man.

## Supporting Information

Text S1 Trial protocol (amendment, French version).
(DOC)

Text S2 Completed CONSORT checklist.
(DOC)
