# Supplementary material for: Praziquantel, Mefloquine-Praziquantel, and Mefloquine-Artesunate-Praziquantel against Schistosoma haematobium: A Randomized, Exploratory, Open-Label Trial
Source: PLoS Negl Trop Dis. 2014 Jul 17;8(7):e2975. doi: 10.1371/journal.pntd.0002975 (PMC4102459; doi:10.1371/journal.pntd.0002975)
Supplement: Text S1 — Trial protocol (amendment, French version). (DOC) [file pntd.0002975.s001.doc]

**Amendement à l’étude, Méfloquine, artesunate et méfloquine-artesunate dans le traitement des infestations à *Schistosoma mansoni* et *S. haematobium* en Côte d’Ivoire”**

L’approbation éthique pour cette étude a été obtenue du Ministère de la Santé et de l’Hygiène Publique (No. 2868; du 4 Juin 2008) et du Comité Ethique de Bâle (Bâle, Switzerland; référence no. 70/08).

Deux essais cliniques exploratoires randomisés, non aveugles ont été conduits entre Octobre et décembre 2008 en zone ouest de Côte d’Ivoire.

Les principales conclusions ont été les suivantes:

- Le plus forts taux de guérison des enfants infestés par *S. haematobium* et *S. mansoni* ont été obtenus avec le praziquantel (88 et 91%).
- Les taux de guérison avec la Méfloquine (25 mg/kg, dose unique), l’artesunate (3 x 4 mg/kg) et la mefloquine-artesunate (3 x (100 mg artesunate + 250 mg méfloquine)) contre les infestations à *S. haematobium* ont été de 21 ; 25 et 61%. Aucune différence statistique significative n’a été observée entre les taux de réduction d’œufs de *S. haematobium* entre les groupes d’enfants ayant reçu le praziquantel d’une part et le mefloquine-artesunate d’autre part (96,4% contre 95,9%). Les taux de réduction des œufs dans les groupes d’enfants ayant reçu la méfloquine et l’artesunate ont été de 73,8 et 84,7% respectivement.
- Les taux de guérison obtenus avec la méfloquine, l’artesunate et la méfloquine-artesunate contre *S. mansoni* ont été 42,1, 25 et 36,8%, respectivement. Chez les enfants infestés par *S. mansoni*, le taux de réduction d’œufs obtenus avec la méfloquine, l’artesunate et la mefloquine-artesunate ont été 87,4 ; 80,3 et 88,6%, respectivement.
- Dans les deux études aucun n’effet secondaire sérieux n’a été rapporté et qui aurait nécessité un recourt à l’hôpital et aucun n’effet indésirable neuropsychologique n’a été observé. Aucun effet indésirable n’a conduit à une discontinuité de l’étude. Les douleurs abdominales ont été le plus fréquent effet secondaire rapporté, légèrement observé chez les enfants traités avec la méfloquine ou la combinaison méfloquine-artesunate que chez ceux traités avec le praziquantel.

Nous aimerions suivre à présent les propriétés antischistosomiase prometteuses de la mefloquine et de la combinaison mefloquine-artesunate. Nous aimerions étudier au cours d’un essai clinique exploratoire randomisé non aveugle l’efficacité des deux (2) combinaisons : praziquantel méfloquine et praziquantel méfloquine-artesunate dans le traitement des infestations à *S. haematobium* et/ou *S. mansoni*. Nous suivrons la même méthodologie utilisée lors de nos deux études antérieures.

- Un consentement éclairé écrit sera obtenu auprès de tous les enfants participant à l’étude.
- 60 enfants (Infestés par *S. haematobium* et/ou *S. mansoni*) seront inclus dans l’étude.
- 20 enfants seront traités avec le traitement standard: une dose orale unique de praziquantel (40 mg/kg).
- 20 enfants seront traités avec le praziquantel (40 mg/kg) suivie de la méfloquine (25 mg/kg, dose unique) et les deux traitements seront espacés de 24 heures.
- Finalement, 20 enfants seront traités avec le praziquantel (40 mg/kg) suivi de la combinaison méfloquine-artesunate (3 x (100 mg artesunate + 250 mg méfloquine) (les deux traitements seront espacés de 24 heures).
- Comme l’élimination de la demi-vie du praziquantel est d’environ 2 heures, il n’y aura pas d’interactions de medicament. Les élèves recevant le traitement seront visités quotidiennement par le médecin de l’étude durant la période d’administration du médicament. Les enfants qui présenteront des effets secondaires seront examinés par le médecin de l’étude et, si besoin, des mesures adéquates seront prises.
- A la fin de l’étude chaque participant à l’étude recevra une dose orale unique de praziquantel 40 mg/kg selon les recommandations de l’OMS. Les enfants infestés par les géohelminthes recevront une dose unique orale d’albendazole 400 mg. Les enfants infestés par *Plasmodium* spp au début de l’étude seront examinés pour l’évaluation de la la parasitémie et s’ils sont positifs, ils seront traités selon les directives nationales relatives à la prise en charge du paludisme.
